# Confirmed and Potential Roles of Bacterial T6SSs in the Intestinal Ecosystem

**DOI:** 10.3389/fmicb.2019.01484

**Published:** 2019-06-28

**Authors:** Can Chen, Xiaobing Yang, Xihui Shen

**Affiliations:** ^1^Institute of Food and Drug Inspection, College of Life Science and Agronomy, Zhoukou Normal University, Zhoukou, China; ^2^State Key Laboratory of Crop Stress Biology for Arid Areas, Shaanxi Key Laboratory of Agricultural and Environmental Microbiology, College of Life Sciences, Northwest A&F University, Yangling, China

**Keywords:** type VI secretion system, intestinal microbiota, enteric pathogen, colonization resistance, competition

## Abstract

The contact-dependent type VI secretion system (T6SS) in diverse microbes plays crucial roles in both inter-bacterial and bacteria-host interactions. As numerous microorganisms inhabit the intestinal ecosystem at a high density, it is necessary to consider the functions of T6SS in intestinal bacteria. In this mini-review, we discuss T6SS-dependent functions in intestinal microbes, including commensal microbes and enteric pathogens, and list experimentally verified species of intestinal bacteria containing T6SS clusters. Several seminal studies have shown that T6SS plays crucial antibacterial roles in colonization resistance, niche occupancy, activation of host innate immune responses, and modulation of host intestinal mechanics. Some potential roles of T6SS in the intestinal ecosystem, such as targeting of single cell eukaryotic competitors, competition for micronutrients, and stress resistance are also discussed. Considering the distinct activities of T6SS in diverse bacteria residing in the intestine, we suggest that T6SS research in intestinal microbes may be beneficial for the future development of new medicines and clinical treatments.

## Introduction

The type VI secretion system (T6SS) is a contact-dependent transmembrane nanomachine that uses a contractile mechanism to inject effectors into adjacent prokaryotic or eukaryotic cells (Hood et al., 2010; Jani and Cotter, 2010; Cianfanelli et al., 2016). A typical T6SS apparatus is composed of 13 core subunits (TssA-TssM) and possesses a structure similar to that of a T4 bacteriophage tail (Boyer et al., 2009; Cascales and Cambillau, 2012; Zoued et al., 2013). In an integral T6SS apparatus, the baseplate complex (TssAEFGK) is anchored to the cell membrane by the membrane complex (TssJLM), providing structural support. The needle sheath (TssBC), tail tube (TssD/Hcp), and spike complex (TssI/VgrG) form the injection apparatus, which is responsible for effector secretion (Cascales and Cambillau, 2012; Silverman et al., 2012). A dynamic T6SS secretion process contains assembly/extension, contraction/puncture, and disassembly of the sheath. Briefly, the membrane complex is formed at the initial stage. Subsequently, baseplate proteins are recruited for sheath/tube anchoring and extension. When attacking, the membrane-puncturing spike pierces target cells through sheath contraction, accompanied by energy consumption and load transportation. Consequently, various effectors are delivered into target cells in a one-step manner (Basler et al., 2012; Brunet et al., 2015; Cianfanelli et al., 2016; Coulthurst, 2019). Although some pathogen-associated T6SSs have been reported to be involved in bacterial pathogenesis (Pukatzki et al., 2007; Aubert et al., 2016; Jiang et al., 2016), the primary function of T6SSs is to compete against rival bacteria in polymicrobial environments (Durand et al., 2014; Cianfanelli et al., 2016). However, other functions of T6SSs, such as resistance to amoeba predation (Zheng et al., 2011), biofilm formation (Gallique et al., 2017), and stress response (Weber et al., 2009; Zhang W. et al., 2013), have also been reported.

The intestinal ecosystem is composed of a densely populated community of multiple microorganisms that may be beneficial or harmful to host health (Walter and Ley, 2011). In intestinal environments where nutrients and space are limited, microbes must use various strategies to coexist or compete with the host and other bacteria. In recent years, the contact-dependent T6SS nanomachine has attracted much attention for its role in interbacterial competition in the mammalian gut (Sana et al., 2017; Verster et al., 2017). T6SS mediated antagonism in the mammalian gut not only benefits the microbiota-mediated colonization resistance by preventing invasion of pathogens, but also assists some pathogens to battle with the resident microbiota to invade an ecosystem and cause disease. Thus, it was stated that there is secret bacterial warfare in the gut, due to the T6SS roles both in the intestinal commensal microbes and enteric pathogens (Sana et al., 2017; Garcia-Bayona and Comstock, 2018). In this review, we have discussed recent studies on intestinal microorganisms with functional T6SS clusters and T6SS-dependent activities related to the intestinal microbial community and host health.

## T6SS-Dependent Activities in Intestinal Ecosystem

### Interference Competition Mediated by T6SS

Bacterial competition occurs as interference competition (killing of target cells with antibacterial weapons) or exploitation competition (consuming nutrients from the milieu) (Chassaing and Cascales, 2018). As an anti-bacterial weapon, T6SSs were primarily considered to mediate interference competition in the intestinal microbiota. The antibacterial function of T6SSs relies primarily on injection of “anti-bacterial” effectors that target conserved, essential features of the bacterial cell, such as peptidoglycan (Russell et al., 2011), membrane phospholipids (Russell et al., 2013), nucleic acids (Ma L.S. et al., 2014), NAD^+^ (Whitney et al., 2015), and the critical cell division protein FtsZ (Ting et al., 2018).

Commensal bacteria form a protective barrier that protects the host from bacterial pathogens (Belkaid and Hand, 2014). In healthy adult individuals, Bacteroidales are the most abundant order of bacteria in the intestinal microbiota (Faith et al., 2013). Recently, by performing extensive bioinformatics analyses and creating hidden Markov models for Bacteroidales Tss proteins, Coyne and colleagues identified 130 T6SS loci within 205 human gut Bacteroidales genomes. As Bacteroidales comprise approximately 50% of all colonic bacteria in many people, this suggested that T6SSs are distributed in about 25% of bacteria in the human colon (Coyne et al., 2014; Coyne et al., 2016). Moreover, more than 10^9^ T6SS-firing events were determined per minute per gram of colonic contents, further supporting the importance of this weapon in shaping gut microbiota composition (Wexler et al., 2016). T6SS loci of the human gut Bacteroidales species segregate into three distinct genetic architectures (GA), termed GA1-GA3. GA1 and GA2 loci are present on conserved integrative conjugative elements (ICE) and are transferred between diverse human gut Bacteroidales strains via ICE. GA3 loci are not contained on conserved ICE and are confined to *Bacteroides fragilis* (Coyne et al., 2014, 2016). The GA3 T6SSs of *B. fragilis* antagonizes human gut Bacteroidales species *in vivo* using previously undescribed effectors, likely to create a locally protected niche in the human gut (Wexler et al., 2016). The interbacterial interactions among symbiotic Bacteroidales species could be predicted according to the presence or absence of strain-specific effector/immunity (E/I) repertoires. Further, some of these strains may avoid contact-dependent killing by accumulating immunity genes to neutralize antibacterial effectors that they do not encode to persist in the gut (Wexler et al., 2016). Importantly, symbiotic non-toxigenic *B. fragilis* strains could restrict enteric colonization by an enterotoxigenic *B. fragilis* strain, dependent on a functional T6S, to protect the host against intestinal inflammatory disease, suggesting a novel role of T6SS in colonization resistance (Hecht et al., 2016; Casterline et al., 2017).

Type VI secretion systems are widely distributed and have been proposed to be present in about 25% of all sequenced Gram-negative bacteria (Boyer et al., 2009; Salomon and Orth, 2015), including enteric pathogenic microorganisms, e.g., *Vibrio cholerae*, *Salmonella enterica*, *Shigella sonnei*, and *Yersinia pseudotuberculosis.* Thus, T6SS mediated antagonism in the mammalian gut both benefits microbiota-mediated colonization resistance by preventing pathogen invasion and facilitates some pathogens to battle resident microbiota to invade the ecosystem and cause disease. Some enteric pathogens could utilize T6SS-mediated antibacterial weapons to kill symbionts and establish within the host gut. For example, *S. enterica* Serovar Typhimurium uses T6SS to kill commensal *Klebsiella oxytoca* and enhance colonization of the mouse gut (Sana et al., 2016). With a functional T6SS, *S. sonnei* showed an advantage in competing against *E. coli* and *Shigella flexneri* both *in vitro* and *in vivo*, which may explain the dominance and increasing global prevalence of *S. sonnei* in developed countries worldwide (Anderson et al., 2017). Constitutive T6SS expression provides *V. cholerae* with an advantage in intra-specific and inter-specific competition, such that T6SS-dependent toxicity toward other bacteria could enhance *V. cholerae* survival in the environment and/or during colonization of a host (MacIntyre et al., 2010; Unterweger et al., 2012; Fu et al., 2013). Through transcriptome sequencing (RNA-Seq), T6SSs and their associated toxins in the gut symbiont of 28 *Snodgrassella alvi* strains from diverse *Apis* and *Bombus* species were analyzed. T6SS-associated Rhs toxins with antibacterial activity could mediate both intraspecific and interspecific competition among *S. alvi* strains and other bee gut microbes. Furthermore, extensive recombination and horizontal transfer of toxin/immunity genes between strains in the gut microbiota have resulted in tremendous diversity in their toxin repertoires, which suggest that T6SS-mediated competition may be an important driver of coevolution (Steele et al., 2017). These studies showed that enteric pathogens could use their T6SSs for interbacterial competition *in vivo* and for niche occupancy.

Besides bacterial competitors, T6SS was also found to target some single cell eukaryotic competitors, including amoebae and fungi. In fact, T6SS was first identified by screening *V. cholerae* mutants that failed to kill the social amoeba *Dictyostelium discoideum*, and the lipid-binding effector VasX was found to be required for T6SS-mediated amoeba killing, possibly through plasma membrane perturbations (Pukatzki et al., 2006; Miyata et al., 2011; Zheng et al., 2011). T6SSs in *Pseudomonas syringae* and *Serratia marcescens* were reported to be required for competition against other single cell eukaryotic organisms, i.e., yeast and fungi (Haapalainen et al., 2012; Trunk et al., 2018). The first anti-fungal T6SS effectors, Tfe1 and Tfe2, were identified in *S. marcescens*. Tfe1 causes plasma membrane depolarization without formation of a specific pore, while Tfe2 disrupts nutrient uptake and amino acid metabolism, and induces autophagy (Trunk et al., 2018). Since single cell eukaryotes are important components of the intestinal microbiota (Coyne and Comstock, 2019), it is not surprising that intestinal bacteria might deploy anti-eukaryotic T6SSs to compete for nutrients and space against co-habiting microbial eukaryotes.

### Exploitation Competition Meditated by T6SS

As essential micronutrients are involved in a wide range of cellular processes (e.g., DNA replication, respiration, and energy generation), transit of these metal ions is a crucial component of host-microbe interactions, including those in the gastrointestinal tract (Sorbara and Pamer, 2019). For example, *E. coli* mutants defective in catecholate siderophore production show impaired murine gut colonization (Pi et al., 2012). Without a high-affinity zinc transporter, *Campylobacter jejuni* is unable to replicate or colonize the gastrointestinal tract, as quantities of zinc in the gastrointestinal tract are reduced by the host (Gielda and DiRita, 2012). Thus, competition for micronutrients in the gut is intense.

Type VI secretion system has been reported to be involved in acquisition of essential micronutrients, such as zinc, manganese, and iron, indicating its novel function in increasing bacterial fitness through competition for essential nutrients. In *Pseudomonas aeruginosa*, the H3-T6SS secreted effector TseF facilitates iron acquisition by interacting with the iron-chelating molecule PQS (Pseudomonas quinolone signal) (Lin et al., 2017). In *Burkholderia thailandensis*, T6SS4 secretes zinc- and manganese-scavenging proteins to fulfill the increased cellular demand for these metal ions under oxidative stress (Si et al., 2017a,b). Similarly, in the enteric pathogen *Y. pseudotuberculosis*, T6SS4 functions to combat host nutritional immunity and multiple adverse stresses by translocating a zinc binding effector YezP (Wang et al., 2015). Distinct from the extensively studied contact-dependent “anti-bacterial” and “anti-eukaryotic” T6SSs, the “metal ion transporting” T6SS secretes metal-binding proteins into the extracellular milieu, independent of cell-cell contact (Si et al., 2017a). Thus, T6SS confers survival advantages to bacterium in niches with multiple bacterial species not only by delivering “anti-bacterial” toxins to kill competing cells for interference competition, but also by enhancing its ability to acquire essential micronutrients for exploitation competition. It will be interesting to determine the physiologic consequence of these metal acquisition T6SSs in the intestinal ecosystem in the future.

Interestingly, expression of *Y. pseudotuberculosis* T6SS4 was found to be nutrient status-dependently regulated by the stringent response factor RelA and the nutrient-dependent regulator RovM (Song et al., 2015; Yang et al., 2019). Further research is needed to reveal whether T6SS is involved in competition for other nutrients besides metal ions.

### Activation of the Innate Immune Response and Virulence

Although it is well-known that enteric pathogens could utilize the antibacterial T6SS weapon to eradicate competing microbes *in vivo*, little is known about whether the T6SS-dependent interactions with commensal bacteria have additional effects on virulence. Recently, Zhao et al. (2018) discovered that the *V. cholerae* T6SS could compete against commensal *E. coli* strains in the mouse gut to facilitate colonization. Importantly, they found that this microbial antagonistic interaction could improve fitness of *V. cholera* by activating host innate immune responses and improving expression of bacterial virulence genes (i.e., cholera toxin) (Zhao et al., 2018). The authors suggested that the innate immune response was induced by bacterial debris derived from lysed commensals, which together with up-regulated cholera toxin (CT), resulted in elevated disease symptoms and increased fitness of *V. cholerae* as a pathogen. Fast et al. (2018) used the *Drosophila melanogaster* model of cholera to define the contribution of T6SS to *V. cholerae* pathogenesis. They found that interactions between T6SS and the gut commensal *Acetobacter pasteurianus* intensified disease symptoms and accelerated host death. The disease severity was attenuated by inactivation of T6SS, or removal of *A. pasteurianus.* Interestingly, mutation of the Immune Deficiency (IMD) pathway relieved T6SS dependent lethality, implicating innate defenses in T6SS-mediated host death (Fast et al., 2018). These studies established that interactions between T6SS and commensal bacteria activate innate immune responses and promote *V. cholera* pathogenesis.

In addition, a few T6SS toxic effectors result in metabolic disorders or diseases in eukaryotic host cells. VgrG-1 in *V. cholerae* was responsible for T6SS-dependent cytotoxicity of macrophages through covalent cross-linking of host cell actin (Pukatzki et al., 2007; Ma and Mekalanos, 2010). In the diarrheal isolate *Aeromonas hydrophila* SSU, T6SS-secreted VgrG1 exhibited actin ADP-ribosylating activity, which can induce a rounded phenotype in HeLa cells, resulting in apoptosis (Suarez et al., 2010). A non-VgrG T6SS effector in *Edwardsiella tarda*, EvpP, inhibited NLRP3 inflammasome activation through repression of the Ca^2+^-dependent MAPK-Jnk pathway (Chen et al., 2017). T6SS effectors with phospholipase activity could cause damage to the cell membrane and accelerate the infection process (Dong et al., 2013; Russell et al., 2013). Notably, a few bacterial pathogens with the T6SS apparatus may spread through the digestive tract but cause diseases outside the gastrointestinal system. For example, in the newborn meningitis *E. coli* (NMEC) K1 strain, the Hcps of T6SS interacts with human brain microvascular endothelial cells (HBMEC) in a coordinated manner, first binding, then invading, and finally causing apoptosis of HBMEC (Zhou et al., 2012). The T6SS-secreted proteins VgrG1 and VgrG2 in *Helicobacter hepaticus* increases cellular innate pro-inflammatory responses, resulting in liver disease and intestinal inflammation (Bartonickova et al., 2013).

### Modulation of Host Intestinal Mechanics

Recently, Logan and colleagues used a combination of microbial genetics, *in vitro* experiments, and quantitative *in vivo* imaging in zebrafish to determine the role of *V. cholerae* T6SS in gut colonization. They found that *V. cholerae* can expel resident microbiota of the genus *Aeromonas* in a T6SS-dependent manner. Unexpectedly, T6SS acted primarily to increase the strength of gut contractions, rather than killing the bacterial competitor. Coupling of T6SS activity to host contractions depended on an actin cross-linking domain (ACD) from the T6SS apparatus. When the ACD domain was deleted, *V. cholerae* could no longer induce enhanced host contractions, and dense *Aeromonas* communities remained in the gut. Deleting the ACD domain did not affect the ability of *V. cholerae* to kill *A. veronii in vitro* or enter and occupy the host intestine. These findings reveal a novel strategy by which enteric pathogens can modulate host intestinal mechanics to redefine gut communities and highlights the role of the host fluid-mechanical environment in shaping gut population dynamics (Logan et al., 2018). Whether this is a common tactic for gut-colonizing bacteria to invade the intestinal ecosystem especially in human needs to be verified in the future.

### Stress Resistance

Within the intestinal lumen, enteric pathogens encounter severe stresses, such as bile salts, antimicrobial peptides, free fatty acids, enhanced osmolarity, and oxidative stress (Fang et al., 2016). To survive these adverse environments, bacteria have developed various delicate mechanisms. As a versatile molecular machine, T6SS has been found to play crucial roles in the bacterial stress response and contribute to cell survival under multiple environmental challenges. *Vibrio anguillarum* T6SS is regulated by the general stress response regulator RpoS and is involved in its resistance to hydrogen peroxide, ethanol, and low pH (Weber et al., 2009). Upon activation by RpoS and the global oxidative stress regulator OxyR, enterohaemorrhagic *Escherichia coli* (EHEC) uses its T6SS to secrete a Mn-containing catalase, KatN, thus providing a higher resistance to reactive oxygen species (ROS) produced by the host and enhancing survival levels in the host (Wan et al., 2017). *Y. pseudotuberculosis* T6SS4 was required for bacterial survival under osmotic, oxidative, and acidic stress and for resistance to bile salts, and its expression was regulated by various stress response regulators, such as the stationary growth phase stress σ factor RpoS, the global oxidative stress regulator OxyR, and the acid/osmotic regulator OmpR (Gueguen et al., 2013; Zhang W. et al., 2013; Guan et al., 2015; Wang et al., 2015). In *V. cholerae* O1, activation of T6SS has been shown to be dependent on the osmolarity of the milieu (Ishikawa et al., 2012). In addition, in the intestine, *V. cholerae* T6SS genes are overexpressed under mucin (intestinal host factors) and microbiota-modified bile salt conditions (Bachmann et al., 2015). Bile salts can also activate expression of T6SS genes in *Salmonella typhimurium* (Sana et al., 2016) and *C. jejuni* (Lertpiriyapong et al., 2012). These studies on gene expression regulation suggest that T6SS is expressed in the intestine and could provide resistance to multiple stresses, increasing bacterial adaptability or survival in the intestinal ecosystem.

### Other Potential Functions

Recently, it was suggested that T6SS in *V. cholerae* serves as a predatory weapon and fosters horizontal gene transfer. For incorporation of DNA released by lysed cells into a competent predator cell, natural transformation and evolution can occur (Borgeaud et al., 2015). Thus, T6SS may promote co-evolution of bacterial microflora within their environments, including intestinal ecosystems.

Compelling data showed that *H. hepaticus* interacts with intestinal epithelial cells (IECs) and employs T6SS to limit within-host growth and virulence (Chow and Mazmanian, 2010). This feature was regarded as T6SS function of anti-virulence (Jani and Cotter, 2010). Nevertheless, this phenomenon is rarely seen.

**FIGURE 1:**
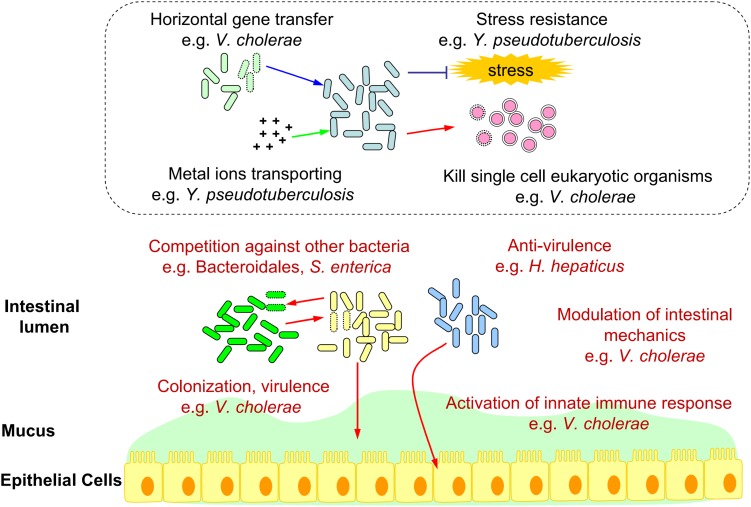
Schematic diagram of bacterial T6SS functions in the intestinal ecosystem. T6SS functions verified in the gut (e.g., Competition against other bacteria, Colonization, Virulence, Anti-virulence, Modulation of intestinal mechanics, and Activation of innate immune response.) were marked in red. Deduced T6SS functions in the gut (e.g., Horizontal gene transfer, Metal ions transporting, Stress resistance, and Kill single cell eukaryotic organisms.) based on *in vitro* analysis were shown in the dotted box.

To summarize, we constructed a schematic diagram showing the functions of T6SS in the intestinal ecosystem (Figure 1).

## Commensal Intestinal Microbes With T6SS Clusters

### Bacteroidetes

Bacteroidetes, a phylum of Gram-negative bacteria, is highly abundant in the gastrointestinal tracts of humans and other mammals (Lozupone et al., 2012; Russell et al., 2014; Wexler and Goodman, 2017). Despite Bacteroidales being predominant in the mammalian gut, T6SSs in Bacteroidetes species were not identified until 2014, perhaps because the T6SS in Bacteroidetes does not have sufficient sequence similarity with core T6SS proteins of Proteobacteria for protein-protein comparisons (e.g., BLASTP) or protein-profile comparisons (e.g., Pfam, COG) (Russell et al., 2014; Coyne et al., 2016). Studies have revealed that the T6SS loci are segregated into three distinct genetic architectures (GA1-GA3), among which the T6SS loci GA1 and GA2 are contained on highly similar integrative conjugative element (ICE) (Coyne et al., 2016). Among the numerous mechanisms that compete in the extremely dense ecosystem of the gut, T6SSs are probably very prevalent antagonistic systems in gut Bacteroidales (Wilson et al., 2015; Chatzidaki-Livanis et al., 2016; Coyne et al., 2016; Coyne and Comstock, 2019), as the Bacteroides T6SS genes are widespread in human gut metagenomes (Verster et al., 2017). Given that T6SSs in Bacteroidetes mediate inter-bacterial antagonism against pathogens and in view of the observed stability of Bacteroidetes in the healthy human intestine, it predicts that T6SS in the stable Bacteroidetes community contributes to intestinal homeostasis. We believe that further research on T6SSs in Bacteroidales species will add to our understanding of microbial stability in the gut and to our ability to diagnose and treat gut microbial infections.

## Enteric Pathogens With T6SS Clusters

### Vibrio cholerae

*Vibrio cholerae* is a Gram-negative pathogen consisting of over 200 serogroups that usually cause diarrhoeal diseases ranging from cholera to mild gastroenteritis after ingestion of contaminated food or water containing them (Kitaoka et al., 2011; Unterweger et al., 2014). All *V. cholerae* strains examined to date contain T6SS gene clusters (Unterweger et al., 2012). This microorganism provided several important and initial discoveries about T6SS, including the definition of the contact-dependent secretion model (Pukatzki et al., 2006; Basler et al., 2012), identification of T6SS-dependent effector-immunity pairs (Dong et al., 2013; Russell et al., 2013; Altindis et al., 2015), and induction of intestinal inflammation through actin cross-linking in host cells (Ma and Mekalanos, 2010). Recently, *in vivo* analysis presented new insight on the pathogenic mechanism of *V. cholerae* with T6SS. T6SS in *V. cholerae* acts on commensal bacteria *A. pasteurianus* to accelerate death of the *Drosophila* host (Fast et al., 2018). Another study revealed the roles of *V. cholerae* T6SS in antagonism against host commensal microbiota *in vivo* over their niche, which facilitated bacterial colonization of the mice gut (Zhao et al., 2018). Besides killing host gut bacterial symbionts, *V. cholerae* T6SS could modulate host intestinal mechanics, enhancing intestinal movements that led to expulsion of resident microbiota by the host (Logan et al., 2018).

### Vibrio parahaemolyticus

Research indicated that vpT6SS2 contributed to adhesion of *V. parahaemolyticus* to host cells (Yu et al., 2012). In addition, the vpT6SS2 effector could induce autophagy in macrophage cells without causing apparent cytotoxicity (Yu et al., 2015).

### Salmonella enterica

Experiments suggested that T6SS contributed to pathogenicity, at least in the *S. enterica* serotypes Gallinarum (Blondel et al., 2013), Enteritidis (Blondel et al., 2010; Troxell, 2018), Typhi (Wang et al., 2011), Dublin (Pezoa et al., 2014), and Typhimurium (Mulder et al., 2012). A recent study provided *in vivo* evidence that *S. typhimurium* used T6SS as a weapon to kill commensal bacteria (*K. oxytoca*) to successfully colonize the mouse gut (Sana et al., 2016).

### Yersinia pseudotuberculosis

Studies of *Y. pseudotuberculosis* revealed roles for T6SS in responses to various stressors and identified corresponding regulators, including OmpR (Gueguen et al., 2013; Zhang W. et al., 2013), OxyR (Wang et al., 2015), ZntR (Wang et al., 2017), RpoS (Guan et al., 2015), RovM (Song et al., 2015), and YpsI/YtbI (Zhang et al., 2011). Notably, T6SS4 in *Y. pseudotuberculosis* was found to be involved in zinc transportation by secreting a zinc-binding protein YezP to mitigate the impact of detrimental hydroxyl radicals under oxidative stress (Wang et al., 2015). The contribution of T6SS to ion transport was also confirmed in *B. thailandensis* and *P. aeruginosa* (Lin et al., 2017; Si et al., 2017a,b).

### Escherichia coli

*Escherichia coli* are model bacteria that have been studied extensively. Although less abundant than Bacteroidales in the gastrointestinal tract, symbiotic gut *E. coli* play important roles in colonization resistance against enteric pathogens of the proteobacterial phylum (Coyne and Comstock, 2019). To date, more than 150 *E. coli* strains have been identified. Most are harmless, but several serotypes have been proposed to be pathogenic and may cause significant diarrheal and extraintestinal diseases (Croxen et al., 2013). The current knowledge regarding the prevalence, the assembly, the regulation, and the roles of the T6SS in *E. coli* has been reviewed (Journet and Cascales, 2016). It has been suggested that T6SS contributes to pathogenesis in *E. coli* strains. For example, T6SS-dependent Hcp1 in *E. coli* K1 induced actin cytoskeleton rearrangement, apoptosis, and release of interleukin-6 (IL-6) and IL-8 in human brain microvascular endothelial cells (HBMEC) (Zhou et al., 2012). Further, T6SS in avian pathogenic *E. coli* (APEC) strains contributes to APEC pathogenesis (Ma et al., 2018). Several structural proteins of T6SS in *E. coli* have been studied to clarify the specific functions of these T6SS components (Felisberto-Rodrigues et al., 2011; Aschtgen et al., 2012; Durand et al., 2012). However, whether *E. coli* gut symbionts have T6SSs that function in colonization resistance against enteric pathogens, and how enteric pathogenic *E. coli* T6SSs function in the intestinal ecosystem still remain enigmatic.

Notably, the T6SS apparatus has a common evolutionary origin with phage tail-associated protein complexes. Yet, the involvement of phages in the evolution of bacterial T6SS remains unclear.

## Other Enteric Pathogens

Type VI secretion system in some other enteric pathogens has also been studied. In *C. jejuni*, T6SS mediates host cell adhesion, invasion, colonization, and adaptation to deoxycholic acid (DCA) (Lertpiriyapong et al., 2012). In *Citrobacter freundii*, T6SS plays a wide-ranging role in the regulation of the flagellar system and in motility; it is also involved in the adherence and cytotoxicity to host cells (Liu et al., 2015). T6SS2 in *Vibrio fluvialis* is associated with anti-bacterial activity and contributes to bacteria survival in highly competitive environments (Pan et al., 2018). The T6SS effector VgrG1 from *A. hydrophila* induces host cell toxicity by ADP ribosylation of actin (Suarez et al., 2010). In *E. tarda*, the T6SS effector EvpP significantly inhibits NLRP3 inflammasome activation by inhibiting the Ca^2+^-dependent MAPK-Jnk pathway (Chen et al., 2017). Additionally, functional T6SSs have been verified in the enteric pathogens *Enterobacter cloacae* (Whitney et al., 2014), *Edwardsiella ictaluri* (Rogge and Thune, 2011), *Yersinia enterocolitica* (Jaakkola et al., 2015), and *V. anguillarum* (Weber et al., 2009). To facilitate retrieval for future research, we have listed the experimentally verified intestinal bacteria that contain functional T6SS loci in Table 1.

**TABLE 1:** Intestinal bacteria with functional T6SSs that have been verified experimentally.

**Bacteria species**	**No. of T6SS loci**	**Experimental strains**	**T6SS associated phenotypes**	**References**
*Aeromonas hydrophila*	1	SSU	Induce host cell toxicity by ADP ribosylation of actin	Suarez et al., 2010
*Bacteroides fragilis*	1	TM4000, ATCC43858, ATCC43859	Virulence and strain competition	Casterline et al., 2017
		NCTC 9343, 638R	Competition	Chatzidaki-Livanis et al., 2016
		NCTC 9343	Interbacterial antagonism	Russell et al., 2014
*Campylobacter jejuni*	1	subsp. *jejuni* serotype O:3	Host cell adhesion, invasion, colonization; adaptation to DCA	Lertpiriyapong et al., 2012
		108	Cytotoxicity toward red blood cells	Bleumink-Pluym et al., 2013
*Citrobacter freundii*	1–2	CF74	Adhesion and cytotoxicity to host cells	Liu et al., 2015
*Citrobacter rodentium*	1–2	DBS100[ATCC51459]	Interbacterial killing	Gueguen et al., 2014
*Edwardsiella tarda*	1–2	EIB202	NLRP3 inflammasome inhibition, bacterial colonization *in vivo*	Chen et al., 2017
*Enterobacter cloacae*	1–2	subsp. *cloacae* ATCC13047	Interbacterial competition	Zhang H. et al., 2013
*Escherichia coli*	1–3	K12 W3110, EAEC 17-2	Bacterial growth competition	Brunet et al., 2013
		APEC SEPT362	Pathogenesis	de Pace et al., 2010
		APEC SEPT362	Enhance biofilm formation	de Pace et al., 2011
		RS218	Pathogenicity in HBMEC	Zhou et al., 2012
		TW-XM (O2:K1)	Pathogenic pathways	Ma J. et al., 2014
		EHEC EDL933	Virulence	Wan et al., 2017
		STEC	Virulence	Aas et al., 2018
*Helicobacter hepaticus*	1	ATCC51449	Modulate and exacerbate the innate pro-inflammatory effect	Bartonickova et al., 2013
		ATCC51449	Limiting colonization and intestinal inflammation	Chow and Mazmanian, 2010
*Salmonella enterica*	1–2	Typhimurium LT2	Pathogenesis	Mulder et al., 2012
		Gallinarum NCTC13346, Enteritidis NCTC13349	Chicken colonization	Blondel et al., 2010
		Typhi GIFU10007	Cytotoxicity of human epithelial cells	Wang et al., 2011
		Typhimurium 14028s	Chicken colonization	Blondel et al., 2013
		Dublin CT_02021853	Colonization of mice and chickens	Pezoa et al., 2014
		Enteritidis	Virulence	Troxell, 2018
		SL1344	Killing of commensal bacteria, Colonization of the host gut	Sana et al., 2016
*Shigella sonnei*	1	CIP106347	Interbacterial competition, niche occupancy	Anderson et al., 2017
*Vibrio alginolyticus*	1–2	EPGS, MVP01, ATCC33787	Regulated by QS and RpoN	Sheng et al., 2012
*Vibrio anguillarum*	1	NB10 serotype O1	Modulates quorum sensing and stress response	Weber et al., 2009
*Vibrio cholerae*	1	C6706	Microbial antagonistic interaction, intestinal colonization, virulence	Zhao et al., 2018
		O37 strain V52	Virulence, competition	Zheng et al., 2011
		O37 strain V52	Intra- and interspecific competition	Unterweger et al., 2012
		A1552	Foster horizontal gene transfer	Borgeaud et al., 2015
		O37 strain V52	Microbial pathogenesis	Pukatzki et al., 2006
		O37 strain V52	Induces an inflammatory diarrhea	Ma and Mekalanos, 2010
		O37 strain V52	Virulence	Miyata et al., 2011
		Multiple strains	Intraspecific competition	Unterweger et al., 2014
		O37 strain V52	Interspecies competition	MacIntyre et al., 2010
		C6706	Intermicrobial competition	Logan et al., 2018
*Vibrio fluvialis*	2	85003	Environmental survival	Pan et al., 2018
*Vibrio parahaemolyticus*	1–2	HZ	Cell adhesion and cytotoxicity	Yu et al., 2012
		RIMD 2210633	Enhance environmental fitness in marine environments, anti-bacterial activity	Salomon et al., 2013
		HZ	Induce autophagy in macrophages	Yu et al., 2015
*Yersinia enterocolitica*	1	subsp. *enterocolitica* 8081	Encumber bacteria invasion and survival in host cells	Jaakkola et al., 2015
*Yersinia pseudotuberculosis*	4–6	YpIII	Maintain intracellular pH homeostasis	Zhang W. et al., 2013
		IP31758	Survival in high osmolarity, resistance to deoxycholate	Gueguen et al., 2013
		YpIII	Transports Zn^2+^ to combat multiple stresses and host immunity	Wang et al., 2015

## Concluding Remarks

Although the composition and function of the intestinal microbiota has been well documented, the underlying mechanisms of its assembly remain poorly understood. T6SS is a contact-dependent molecular weapon primarily used for interbacterial competition. Considering that T6SS loci exist in 50% of intestinal Bacteroidetes, which are the dominant microflora in the gastrointestinal system, and in some other commensal intestinal microbes and enteric pathogens, T6SS might be widely used in the intestinal ecosystem to shape microbiota composition. Actually, several seminal studies have greatly advanced our understanding of the importance of T6SS in the intestine. These studies showed that the T6SS antibacterial weapons are not only used by pathogens to colonize their hosts, but also by gut commensals to prevent pathogen colonization. Interestingly, two studies in *V. cholera* further indicated that microbial antagonistic interactions mediated by T6SS could elevate disease symptoms by activating host innate immune responses (Fast et al., 2018; Zhao et al., 2018). Moreover, using a zebrafish model, Logan et al. (2018) revealed that the T6SS of *V. cholera* could modulate host intestinal mechanics to redefine the gut microbial composition. These findings provide new insights into the mechanisms used by enteric pathogens for gut colonization. As for the gut commensals, Verster et al. (2017) investigate the prevalence and roles of the T6SS in the human gut microbiome by using metagenomic analyses. In addition to the compatibility with species GA1 and GA2, GA3 is associated with increased Bacteroides abundance in infant microbiomes, and confers an advantage in Bacteroides-rich ecosystems. Thus, it revealed the prevalence and potential role of T6SS-dependent competition in shaping human gut microbial composition (Verster et al., 2017).

It is noteworthy that sometimes a few invertebrate models and small animal models (e.g., Drosophila, zebrafish) were used to analyze T6SS functions in the intestinal ecosystem (Fast et al., 2018; Logan et al., 2018). These results should be further verified in mammalian models. In addition, further *in vivo* studies are urgently needed to confirm whether anti-fungal, stress-resistant and metal ion-acquiring T6SSs are functional in the intestinal ecosystem. Studies of T6SS in the intestinal ecosystem are just beginning and have a long way to go. As T6SS in several bacterial pathogens contributes to virulence against the host, the T6SS apparatus could be exploited as a therapeutic drug target. Based on this possibility, a novel natural antimicrobial was identified that can reduce the pathogenicity of *Campylobacter* T6SS *in vitro* and also decrease its colonization *in vivo* (Sima et al., 2018). These findings suggest that studies on T6SS functions in the intestinal ecosystem may provide a theoretical basis for the development of new medicines for various pathogenic infections in the future.

## Author Contributions

CC and XY collected and assessed the references. CC and XS contributed in the proposal and guidelines of the review. CC and XY wrote this manuscript.

## Conflict of Interest Statement

The authors declare that the research was conducted in the absence of any commercial or financial relationships that could be construed as a potential conflict of interest.
